# Progressive loss of CD3 expression after HTLV-I infection results from chromatin remodeling affecting all the CD3 genes and persists despite early viral genes silencing

**DOI:** 10.1186/1743-422X-4-85

**Published:** 2007-09-06

**Authors:** Haidar Akl, Bassam Badran, Gratiela Dobirta, Germain Manfouo-Foutsop, Maria Moschitta, Makram Merimi, Arsène Burny, Philippe Martiat, Karen E Willard-Gallo

**Affiliations:** 1 Laboratory of Experimental Hematology, Institut Jules Bordet, Université Libre de Bruxelles (ULB), 121, Boulevard de waterloo, 1000, Brussels, Belgium; 2 Molecular Immunology Unit, Institut Jules Bordet, Université Libre de Bruxelles (ULB), 127, Boulevard de waterloo, 1000, Brussels, Belgium

## Abstract

**Background:**

HTLV-I infected CD4^+ ^T-cells lines usually progress towards a CD3^- ^or CD3^low ^phenotype. In this paper, we studied expression, kinetics, chromatin remodeling of the CD3 gene at different time-points post HTLV-I infection.

**Results:**

The onset of this phenomenon coincided with a decrease of *CD3*γ followed by the subsequent progressive reduction in *CD3*δ, then *CD3*ε and *CD3*ζ mRNA. Transient transfection experiments showed that the *CD3*γ promoter was still active in CD3^- ^HTLV-I infected cells demonstrating that adequate amounts of the required transcription factors were available. We next looked at whether epigenetic mechanisms could be responsible for this progressive decrease in CD3 expression using DNase I hypersensitivity (DHS) experiments examining the *CD3*γ and *CD3*δ promoters and the *CD3*δ enhancer. In uninfected and cells immediately post-infection all three DHS sites were open, then the CD3γ promoter became non accessible, and this was followed by a sequential closure of all the DHS sites corresponding to all three transcriptional control regions. Furthermore, a continuous decrease of *in vivo *bound transcription initiation factors to the *CD3*γ promoter was observed after silencing of the viral genome. Coincidently, cells with a lower expression of CD3 grew more rapidly.

**Conclusion:**

We conclude that HTLV-I infection initiates a process leading to a complete loss of CD3 membrane expression by an epigenetic mechanism which continues along time, despite an early silencing of the viral genome. Whether CD3 progressive loss is an epiphenomenon or a causal event in the process of eventual malignant transformation remains to be investigated.

## Background

HTLV-I infection can lead to the development of adult T-cell leukemia/lymphoma (ATLL) in 2–5% of infected individuals depending upon geographic location and exposure to etiologic factors. It is currently thought that tumors develop from a persistently infected T-cell reservoir, which can be amplified by cytokine-induced activation leading to viral gene expression, cellular proliferation and survival of some expanded cells. Viral gene expression has been implicated in the disruption of various normal cellular processes, including activation, growth, and apoptosis, the latter allowing accumulation of abnormalities leading to cellular transformation. Several viral proteins have been shown to play an important role in tumor progression by modulating transcription factors. The pleiotropic viral protein Tax mediates the NF-κB activation resulting in abnormal cytokine and cytokine receptor expression[1]. Sumoylation and ubiquitination of Tax are critical for Tax mediated transcriptional activity[2,3]. The viral protein p12^I ^stimulates calcium release from the endoplasmic reticulum, which induces NFAT transcription factors leading to T-cell activation[4,5]. The viral protein HBZ represses c-Jun mediated transcription by inhibiting its DNA binding activity[6].

A keystone of the antigen-specific immune response is the T-cell receptor (TCR)/CD3 complex. Infected CD4^+ ^lines and T-cells from patients with ATLL are characterized by a CD3^- ^or CD3^low ^phenotype [7-9]. In a previous work[10] we have shown that HTLV-I infected cells acquired a profound decrease of intracellular calcium levels in response to ionomycin, timely correlated with decreased CD7 and CD3 expression. This perturbation induced Akt and Bad phosphorylation via activation of PI3K. The activation of the Akt/Bad pathway generates a progressive resistance to apoptosis, at a time HTLV-I genes expression is silenced. Since dysregulation of calcium flux after T-cell activation has been suggested as a possible consequence of absence of CD3 expression[11]. We decided to investigate the mechanisms responsible for the loss of CD3 expression, its kinetics and its timely relationship with viral gene expression.

Experimental infection of CD4^+ ^T cells with HTLV-I was known to progressively downregulate *CD3 *genes transcripts, eventually leading to a CD3^- ^surface phenotype after 200 days of *in vitro *infection [12,13]; however, the sequence of CD3 genes loss of expression had not been investigated. Previous data from our laboratory showed that CD3 membrane expression was downmodulated after experimental infection of CD4^+ ^T cells with HIV-1 [14-17], HIV-2[18], as well as in patients with CD3^- ^CD4^+ ^T-cell lymphoma mediated hypereosinophilic syndrome [19], all linked to a specific defect in *CD3*γ gene transcripts. All T-lymphotropic viruses induce CD3 downregulation in the absence of a generalized suppression of host protein synthesis.

The HTLV LTR responds to T cell-activation signals[20], which suggests an important relationship between the regulation of viral gene transcription and the TCR/CD3-controlled antigen activation pathway. This study demonstrates that HTLV-I associated loss of CD3 expression is also linked to an initial loss of *CD3*γ gene transcripts, ultimately leading to a CD3^- ^phenotype. However, we show that the initial *CD3*γ transcripts decrease is followed by a subsequent progressive and sequential reduction in *CD3*δ, *CD3*ε and *CD3*ζ genes transcription, going on after early viral genes silencing. Our experiments also demonstrate that these phenomena occur through chromatin remodeling and progressive closure of the CD3 genes promoter sites and are not the results of transcription factors depletion. Finally, this loss of CD3 expression is timely associated with a growth advantage, but further experiments will be needed to determine whether there is a causal relationship between these two observations.

## Methods

### Cell culture conditions and reagents

The WE17/10 cell line is a human IL-2 dependent CD4^+ ^T cell line[14] that was established and is maintained in RPMI 1640 containing 20% fetal bovine serum, 1.25 mM L-glutamine, 0.55 mM L-arginine, 0.24 mM L-asparagine, and 100 units of recombinant human IL-2 per ml. The MT-2 cell line was derived by co-culturing normal umbilical cord leukocytes with donor leukemic T-cells from an HTLV-I infected patient [21]. WE17/10 cells were co-cultured with irradiated MT-2 cells at a ratio of 1:1 to generate HTLV-I infected WE17/10 cell lines. The human B lymphocyte line, GM-607, was obtained from the Human Genetic Cell Repository run by Coriell Institute, Camden NJ). The HTLV-1-transformed T-cell lines (C91-PL, MT-2), were obtained from MT-2, C91-PL and GM-607 cell lines were maintained in RPMI 1640 supplemented with 10% fetal bovine serum and ATL-derived culture (PaBe).

### Southern blot

We used a standard southern blot protocol. The genomic DNA was digested with E*co*RI (no cut into the HTLV-I provirus) or S*ac*I (cut once into the HTLV-I LTR) and electrophoresed in an agarose gel then transferred to nylon membrane (Amersham International, Buckinghamshire, UK). The filters were hybridized with radiolabeled probe : a *Kpn*I fragment[22], corresponding to a 2.9 kb fragment beginning in the *pro *gene and ending in the *env *gene, at 65°C for 12 hours, washed in buffers, and then exposed to X-ray film at -80°C.

### Flow Cytometry

Cells were analyzed for CD3 surface expression by flow cytometry as previously described[17]. Briefly, cells were labeled with the murine monoclonal antibody Leu4a (BD Biosciences, Erembodegen, Belgium) in a two-step process using 1 μg/ml of the primary antibody to ensure saturation binding followed by the manufacturer's recommended dilution of fluorescein-conjugated goat anti-mouse immunoglobulin (BD Biosciences). The labeled cells were fixed in 2% paraformaldehyde, and fluorescence was analyzed on a FACS Caliber (BD Biosciences).

### Transient transfection

WE17/10 cells (uninfected and HTLV-I infected) were transiently transfected using standard DEAE-dextran protocols with wild-type (pHγ3-wt) promoter construct as previously described[17,23].

### Identification of Dnase I hypersensitive sites

Isolation and DNase I digestion of nuclei was performed using a method previously described [24]. Briefly, the cells were washed in PBS and resuspended in cell lysis buffer (60 mM KCl, 15 mM NaCl, 5 mM MgCl_2_, 10 mM Tris pH 7.4, 300 mM sucrose, 0.1 mM EGTA, and 0.1% NP-40) to isolate the nuclei. The nuclei were then resuspended in 1 ml of nuclear digestion buffer (60 mM KCl, 15 mM NaCl, 5 mM MgCl_2_, 10 mM Tris pH 7.4, 300 mM sucrose, and 0.1 mM EGTA). Nuclei from 20 × 10^6 ^cells were digested for 3 minutes at 22°C using increments of DNase I (Roche Diagnostics) from 0 to 28 U/ml. The reaction was stopped by adding nuclear lysis buffer (300 mM sodium acetate, 5 mM EDTA pH 7.4, 0.5% SDS) containing 0.1 mg/ml proteinase K and incubating for 5 min at 55°C then overnight at 37°C. Genomic DNA was subsequently isolated using standard phenol chloroform extraction techniques.

Genomic DNA was digested with BglI for the *CD3*δ promoter, BamHI for the *CD3*δ enhancer and SacI for the *CD3*γ promoter prior to standard Southern blot analysis. Promoter probes were amplified by PCR using the following primer pairs:

*CD3γ promoter: *forward, 5'-CACCTGCTGAAACTGAGCTG-3', reverse, 5'-TCCCAGACAGTGGAGGAGTT-3';

*CD3δpromoter: *forward, 5'-GTTCCTCTGACAGCCTGAGC-3' and reverse 5'-TTTTAGGCCTGATGGCCTCT-3'.

The probe used to detect the *CD3*δ enhancer was a BamHI digest of the human *CD3*δ cDNA (NCBI accession # BC070321).

### RT-PCR

Total RNA was isolated from cells using the TriPure Isolation Reagent (Roche Applied Science) in a single-step extraction method. Standard reverse transcription was performed using 1 μg of total RNA at 42°C for 45 minutes and 50 ng of the resulting cDNA was used per PCR reaction. The primer pairs used to amplify the individual *CD3 *genes have been previously described[25,26] and are as follows:

*CD3*γ: forward 5'-CATTGCTTTGATTCTGGGAACTGAATAGGAGGA-3', reverse 5'-GGCTGCTCCACGCTTTTGCCGGAGACAGAG-3';

*CD3*δ: forward 5'-TTCCGGTACCTGTGAGTCAGC-3', reverse 5'-GGTACAGTTGGTAATGGCTGC-3'.

### Quantitative real-time RT-PCR

Real-time RT-PCR was performed using a TaqMan Gene Expression Assay for each of the individual CD3 genes (CD3ζ HS00609512, CD3ε HS00167894, CD3γ HS00173941 and CD3δ HS00174158; Applied Biosystems, Lennik, Belgium). Eukaryotic translation elongation factor1 α(EF-1-α) and cancer susceptibility candidate 3 (MLN51) were used as CD4+ T cell specific endogenous reference genes as described by Hamalainen *et al*[27]. Relative quantification was used to compare the changes in CD3 mRNA levels using the endogenous genes (EF-1-α and MLN51) as a normalizer and uninfected WE17/10 cells as a calibrator. The individual CD3 genes were normalized to the endogenous controls and the values are expressed as the quantity relative to the uninfected WE17/10 cell line. Biological duplicates were performed for all genes tested.

### EMSA

Nuclear extracts were prepared from 2 × 10^7 ^cells, and EMSA experiments were performed as described previously[17]. The radiolabeled oligonucleotide probe used for nuclear protein binding was an oligonucleotide encoding wild-type Spγ_**1**_/CD3γInr binding site: Spγ_**1**_/CD3γInr_**wt**_, 5'-GTGATGGGTGGAGCCAGTCTAG-3'[23]. The oligonucleotide bound complexes were separated on a 6% Tris-glycine-EDTA polyacrylamide gel migrated overnight at 50 V, and the radiolabeled protein complexes were detected by autoradiography.

### Chromatin immunoprecipitation (ChIP) assay

The ChIP assay was performed as previously described[28] using the kit purchased from Upstate Biotechnology generally following the manufacturer's protocol. Uninfected and HTLV-I-infected WE17/10 cells were fixed with 1.5% formaldehyde for 10 min at 37°C. Chromatin was isolated, sheared using a Bioruptor (Diagenode), and immunoprecipitated with Abs directed to ac-H4, HDAC1, Sp1 (SC-59X), Sp3 (SC-644X), TFIID (SC-204X) (all from Santa Cruz Biotechnology), or control rabbit IgG (Upstate Biotechnology). Cross-linking was reversed by heating, and the proteins were removed subsequently by proteinase K digestion. The presence of selected DNA sequences in the immunoprecipitated DNA was assessed by PCR using the following primer pair Spγ_**1**_, CD3γ_Inr_, and Spγ_**2 **_(205-bp product), forward, 5'-GGGTTCTTGCCTTCTCTCTCAA-3', reverse, 5'-CCCCTAGTAGGCCCTTACCTT-3'.

The amplified ^32^P-labeled PCR product was separated on a 6% acrylamide gel and detected by autoradiography.

## Results

### CD3 loss after HTLV-I infection is linked to a sequential reduction in CD3 gene transcripts

The cell lines were derived from the IL-2 dependent CD4^+ ^T cell line WE17/10 infected by the HTLV-I viruses produced by the MT-2 cell line. The latter, used as virus source, contains 8 complete or defective proviral genomic integrations some defective proviral genomes being able to produce viral RNA transcripts. The most dominant species of unintegrated viral DNA was 3.7 kb in size; it hybridized to a full-length HTLV-1 DNA probe but not to a *KpnI *viral DNA fragment beginning in the *pro *gene and ending in the *env *gene[29] that is absent from a defective proviral genome that has been previously identified in MT-2 cells.

**Figure 1:**
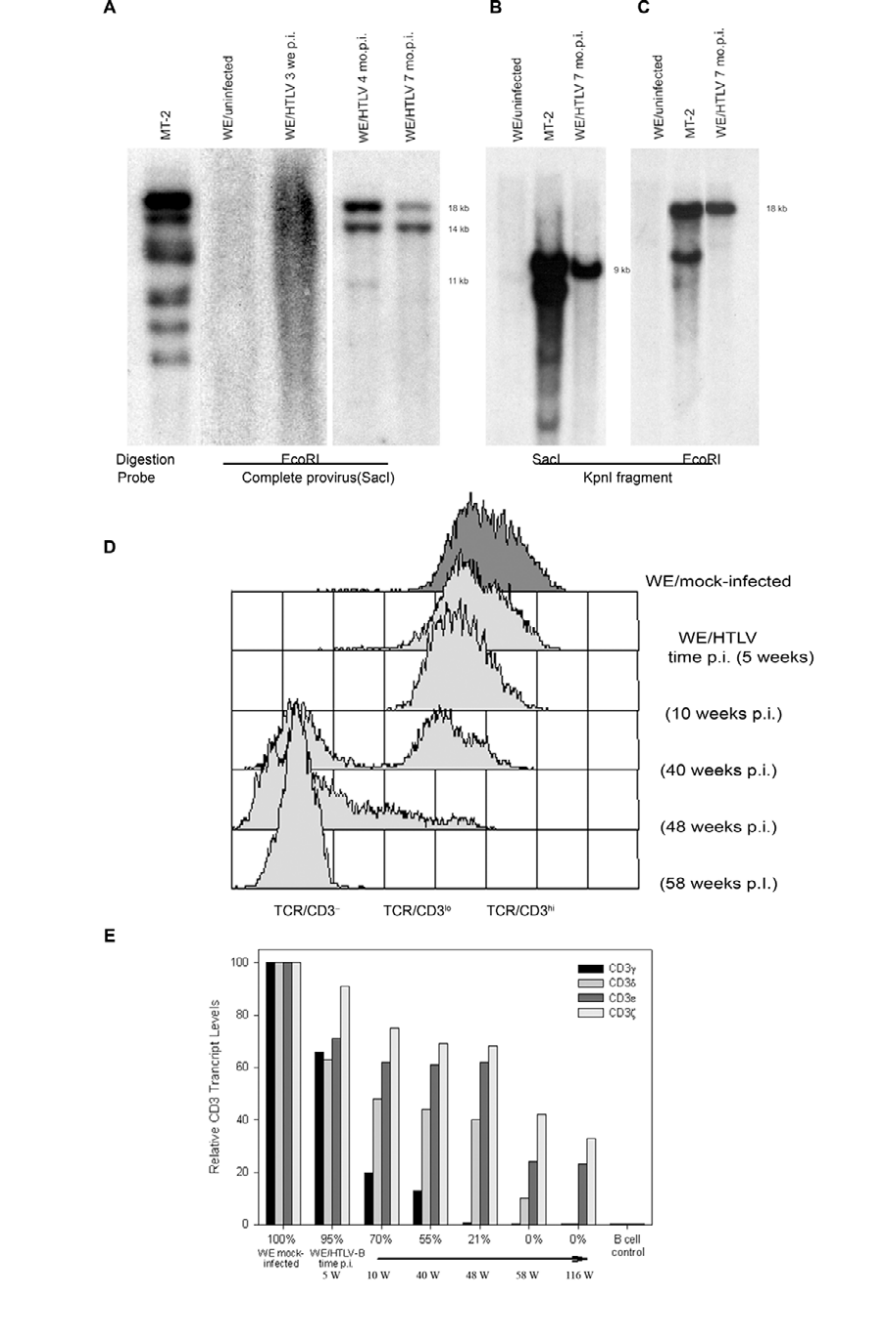
**Proviral integration, CD3 surface expression and relative *CD3 *gene expression over time after HTLV-I infection of WE17/10 cells**. *A*, HTLV-I proviral genome analyses of WE/HTLV cell line by Southern blot. the complete provirus probe was hybridized to the WE/HTLV (at 3 weeks, 4 and 7 months p.i.) genomic DNA digested with E*co*RI. *B*, the K*pn*I fragment probe was hybridized to the (at 7 months p.i.) genomic DNA digested with S*ac*I. *C*, the K*pn*I fragment probe was hybridized to the (at 7 months p.i.) genomic DNA digested with E*co*RI. MT-2 and uninfected WE17/10 cell lines were used as positive and negative control respectively. *D*, TCR/CD3 surface expression over time after HTLV-I infection of WE17/10 cells. profiles showing the distribution of immunofluorescence from anti-CD3 antibody staining in a parallel antibody labeling experiment. Uninfected and HTLV-I infected cells were thawed from the frozen cell line bank at 5, 10, 40, 48, and 58 weeks p.i. TCR/CD3^low ^cells are identified as cells that fall below the minimum fluorescence intensity defined by the positive control but do not lie within the region defined by the negative control. TCR/CD3^hi ^cells fall within the region defined by mock-infected cells, and TCR/CD3^- ^cells fall within the region designated by the negative control. *E*, Histograms representation of relative *CD3 *gene expression in HTLV-I infected cells at various times p.i. determined by real time RT-PCR in relation to the percentage of surface TCR/CD3^+ ^cells determined by flow cytometry. All percentages were calculated relative to uninfected cells (100% positive). GM-607 B cell line was used as a negative control.

At 2 months p.i. using *Eco*RI, which does not cut within the 9 kb of the HTLV-I genome, the complete provirus probe revealed a smear witnessing a polyclonal integration of the provirus in the WE17/10 infected cells (Figure 1A).

At 4 months p.i. the same experiment showed three bands of 18, 14 and 11 kb. At 7 months p.i. Only the 18 an 14 kb bands were evident suggesting at that time a biclonal proliferation of infected cells in the culture. Using the *Kpn*I fragment as probe we detected a 9 kb band when the genomic DNA was digested with S*ac*I, an enzyme cutting once in each HTLV-I LTR (Figure 1B). The same *Kpn*I probe revealed an 18 Kb fragment after E*co*RI DNA digestion (Figure 1C). Our data suggests that a WE17/10 clone, harboring one complete and one incomplete HTLV-I provirus, not detected by the *Kpn*I probe, has a significant growth advantage. This is in accordance with the fast growing cultures observed later on.

ATLL patients are routinely characterized as having a CD3^- ^or CD3^low ^phenotype [7-9]. Experimental infection of CD4^+ ^T cells with HTLV-I and HTLV-II[12,13] has also been associated with defects in TCR/CD3 expression and function. We have tested the HTLV-I infected cell lines MT-2, C91, WE/HTLV and an ATLL derived cell line PaBe for their TCR/CD3 surface expression. All the cells had a CD3^- ^or CD3^low ^phenotype (Additional file 1).

For WE/HTLV we have studied the kinetics of the CD3 surface expression loss. Initially, during the acute phase of infection, cell growth was slowed down by virus production and a significant cytopathic effect. At this time, assessment of TCR/CD3 surface expression by flow cytometry was difficult. Chronically infected cells, appearing around 3 weeks p.i., returned to a normal growth rate and expressed CD3 levels similar to the mock-infected control until 5 weeks p.i., the time when CD3^low ^expressing cells first emerged.

Cryopreserved cells from different stages of the primary infection were thawed and CD3 surface density was quantified in a parallel experiment to ensure that the detected changes were not attributable to variation in antibody labeling experiments (Figure 1D). A significant reduction in CD3 density on the infected cell surface, corresponding to the CD3^low ^phenotype, was detected at 6 to 10 weeks p.i. The cells remained CD3^low ^until receptor negative cells began to emerge around 7 months p.i. followed by the complete loss of surface expression at approximately one year p.i. Thus, CD3 expression on chronically HTLV-I infected cells (WE/HTLV) decreased in a progression from CD3^hi ^to CD3^low ^to CD3^-^, similar albeit slower than that previously described for HIV-infected cells[14,15,18]. The mock-infected cells, carried in parallel passages, continuously maintained CD3^hi ^expression.

A previous study[13] found that all four CD3 chains transcripts (*CD3*γ, δ, ε and ζ) were lost after HTLV-I infection *in vitro*, but these experiments did not provide insight into the order of their loss. Our previous experiments have shown that TCR/CD3 surface receptors are down-modulated after infection with HIV-1[14,17] and HIV-2[18] linked to an initial reduction in *CD3*γ gene transcripts. We therefore asked whether the *CD3*γ gene was also initially targeted after HTLV-I infection and found that its specific decrease of transcription precedes the progressive loss of surface CD3 expression on HTLV-I infected cells.

A real time RT-PCR assay for quantification of all four CD3 gene transcripts revealed that the loss of TCR/CD3 complex at the cell surface occurs quite later than the loss of *CD3*γ transcripts (Figure 1E). Initially, at 5 weeks p.i. there is a 25% decrease in *CD3*γ, *CD3*δ and *CD3*ε transcripts observed in infected cells, shown by flow cytometry to express ~95% TCR/CD3^+ ^surface complexes (relative to the uninfected controls). Subsequently, a precipitous drop of about 80% in *CD3*γ transcripts appears while the density of the TCR/CD3 on the cell surface is ~70%. This erosion in *CD3*γ transcript numbers progresses until all of the cells are *CD3*γ and surface CD3 negative (± 9–12 mo. p.i.). This loss of *CD3*γ gene expression is followed by a steady decrease in *CD3*δ transcripts followed by a slower but also progressive reduction in *CD3*ε and *CD3*ζ transcripts. Maintained continuously *in vitro*, the HTLV-I infected cells eventually become negative for *CD3*δ as well as *CD3*γ transcripts. The level of *CD3*ε and *CD3*ζ transcripts remains ~25% in the CD3γ^-^δ^- ^cells even after more than three years p.i. In MT-2 cells *CD3*γ, *CD3*δ and *CD3*ε transcripts are completely lost while the *CD3*ζ transcripts are still expressed but at a very low level (data not shown).

### The CD3γ promoter can be activated in CD3^- ^HTLV-I infected WE17/10 cells

**Figure 2:**
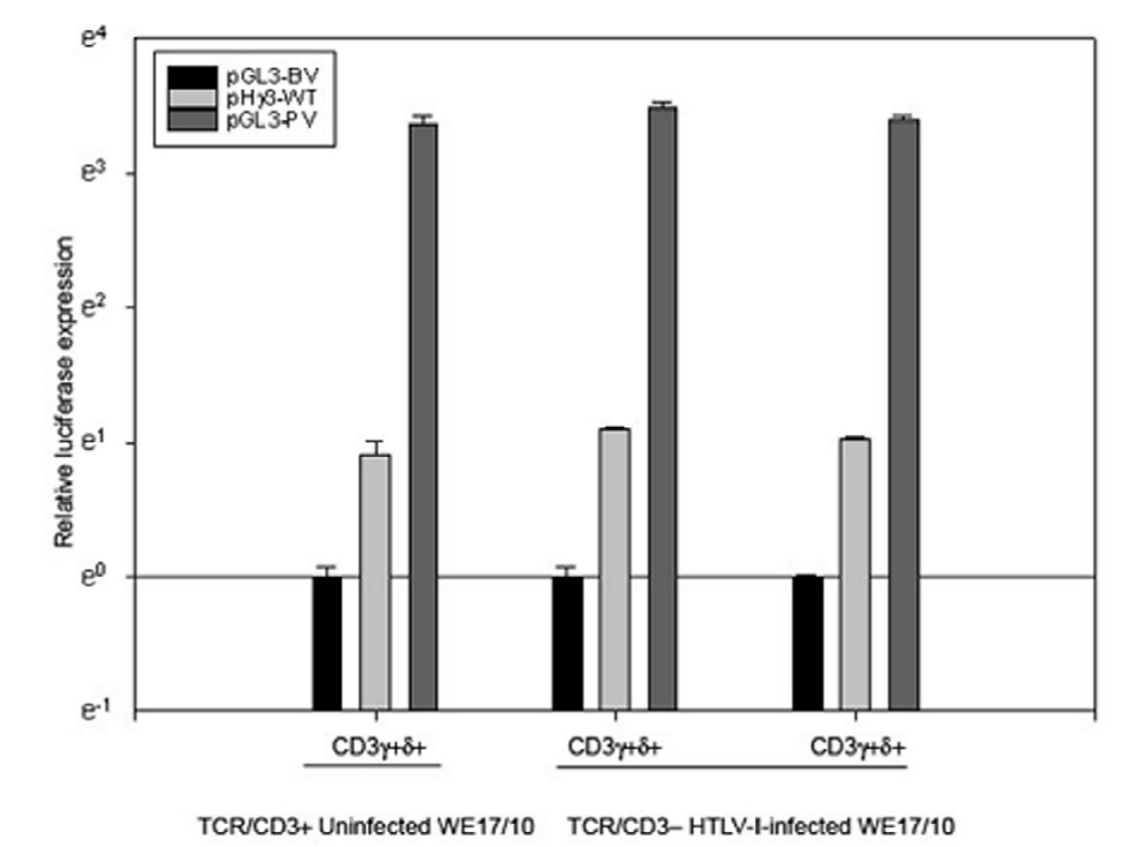
**Functional analysis by transfection of the *CD3*γ promoter activity in HTLV-I infected and uninfected cells**. Luciferase activity was measured in uninfected CD3γ^+^δ^+^, HTLV-I-infected CD3γ^-^δ^+ ^and CD3γ^-^δ^- ^WE17/10 cells after 40 h and normalized to activity from the internal *Renilla *control. Expression of the wild-type *CD3*γpromoter constructs (pH γ3-wt) was measured in comparison to the negative control basic vector: (pGL3-BV) set to one. The pGL3 promoter vector (pGL3-PV) was used as a positive control. The results represent at least three individual experiments, each performed in triplicate.

In an effort to investigate the full-length *CD3*γ promoter activity in the HTLV-I infected cells after the loss of *CD3*γ gene expression we used our previously described construct (pHγ3-wt)[23] in a transient reporter assay (Figure 2). pHγ3-wt was transfected into uninfected and HTLV-I infected WE17/10 cells. Interestingly, in CD3γ-δ+ and CD3γ-δ- HTLV-I infected WE17/10 cells, the *CD3*γ promoter activity was similar to that of uninfected WE17/10 cells. It was over 2.5 fold of the activity measured for the pGL3 plasmid basic vector (pGL3-BV). The *CD3*γ promoter cloned into a plasmid vector was active while the *CD3*γ gene transcripts are lost after HTLV-I infection. Thus, after HTLV-I infection, *CD3*γ gene silencing could not be explained by a lack of transcription factors but potentially by a restrained accessibility to its transcriptional regulation region.

### Chromatin studies: analysis of DNase I hypersensitivity sites in the CD3γ/CD3δ gene region

**Figure 3:**
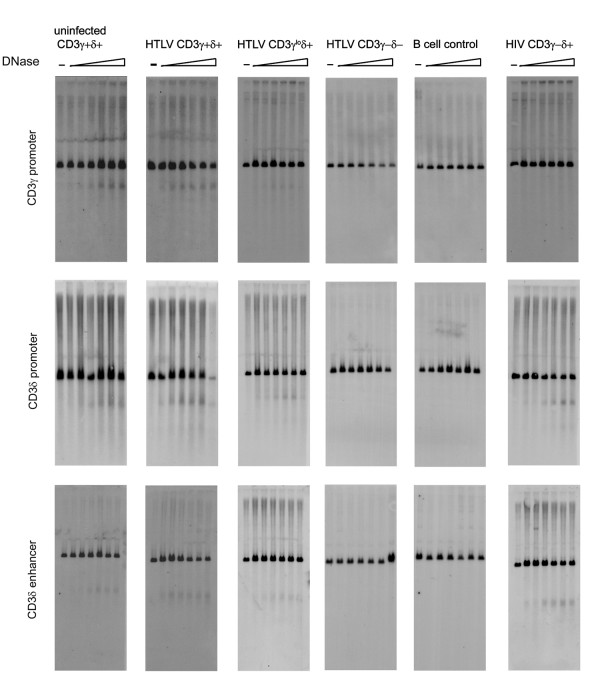
**DNase I hypersensitivity of *CD3*γ and *CD3*δ genes regulatory regions after HTLV-I infection**. DNase I hypersensitivity experiments using probes designed to specifically detect the *CD3*γ promoter, *CD3*δ promoter or *CD3*δ enhancer, indicated on the Y axis. DNA was digested with increasing concentrations of DNase I (increasing from left to right in each panel) and extracted from uninfected CD3γ^+^δ^+ ^cells and HTLV-I CD3γ^+^δ^+^, CD3γ^lo^δ^+^, and CD3γ^-^δ^- ^cells. The B cell (CD3 negative) and HIV-1 CD3γ^-^δ^+ ^cell lines were used as controls. The various cell lines are indicated on the X axis. The level of surface TCR/CD3 expression and relative CD3 gene transcripts for each cell line is shown in Table I.

The human *CD3*γ, *CD3*δ and *CD3*ε genes are located in a 50 kb cluster on chromosome 11q23, with *CD3*γ and *CD3*δ positioned head-to-head and separated by 1.6 kb. DNase I hypersensitivity experiments using probes designed to specifically detect the *CD3*γ promoter, *CD3*δ promoter or *CD3*δ enhancer (an enhancer for the *CD3*γ gene has not been identified yet) revealed that in uninfected (positive control) and HTLV-I infected CD3γ^+^δ^+ ^cells all three DNase I hypersensitive sites (DHS) are readily discernible (Figure 3; relative surface CD3 expression and transcript levels are shown in Table 1). In contrast, in CD3γ^lo^δ^+ ^cells, the *CD3*γpromoter DHS site is weakly detectable while the *CD3*δ promoter and enhancer DHS sites are still clearly evident. In HTLV-I infected CD3γ^-^δ^- ^cells, the DHS sites corresponding to all three transcriptional control regions show no open chromatin in this region similar to the B cell line GM-607 used as a negative control. Taken all together our results suggest a potential chromatin remodeling process taking place after HTLV-I infection associated to the CD3 locus silencing.

**Table 1:** TCR/CD3 expression in cells used for the DNase I hypersensitivity assay

	**Surface TCR/CD3 (flow cytometry)**	**mRNA transcripts (real-time RT-PCR)**	
**Cells**	**CD3^+ ^cells**	**CD3γ**	**CD3δ **

uninfected	100%	100%	100%
HTLV-I γ^+^δ^+^	98%	85%	70%
HTLV-I γ^lo^δ^+^	55%	13%	44%
HTLV-I γ^-^δ^-^	0%	0%	0%
B cell control	0%	0%	0%
HIV-1 γ^-^δ^+ ^control	0%	0%	70%

### Chromatin studies: CHIP experiments

**Figure 4:**
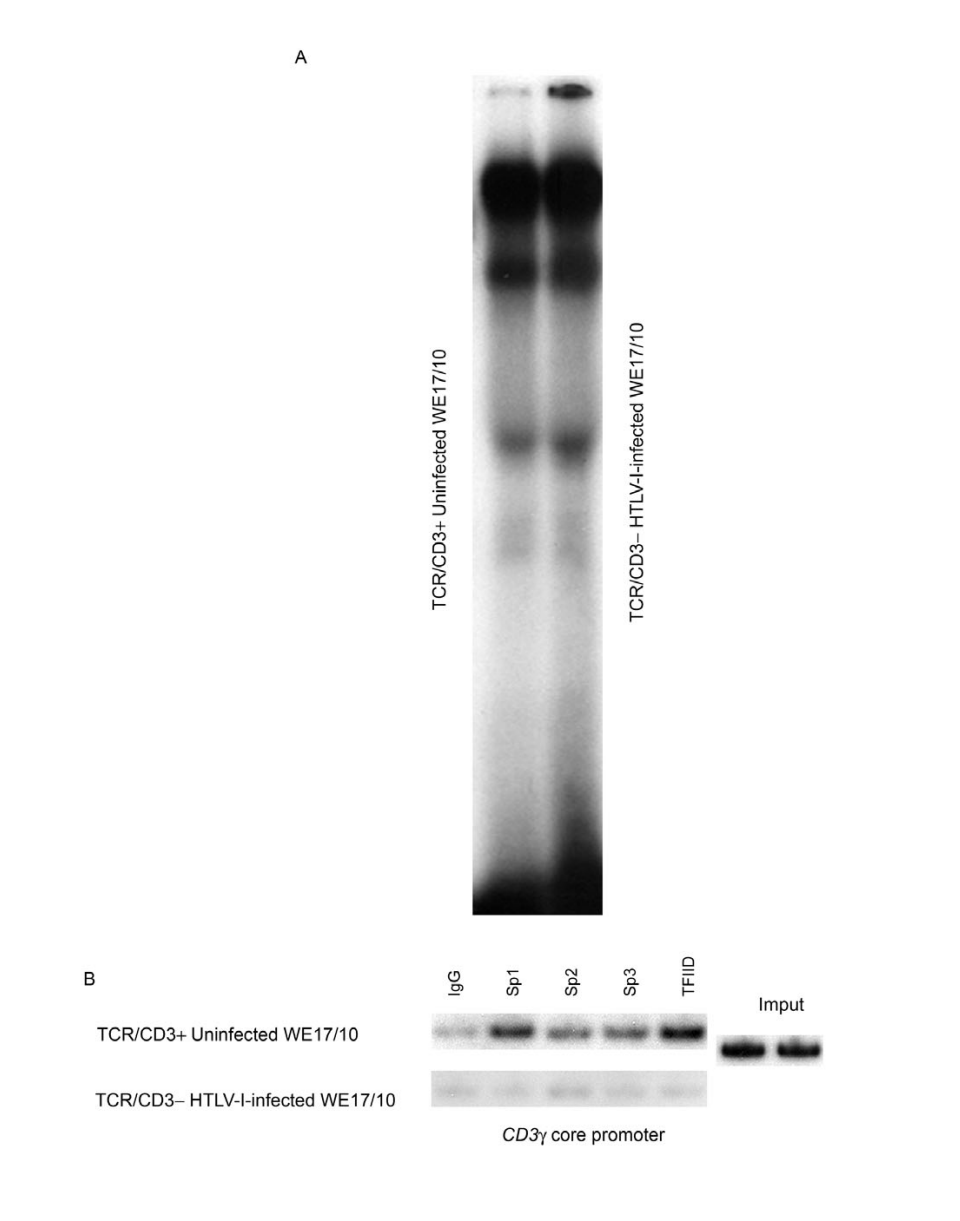
**Transcription factor accessibility to the *CD3*γ promoter after HTLV-I infection**. *A*,*In vitro *binding to the Spγ_**1**_/CD3γ_**Inr **_[22] wild-type probe was examined in EMSA assay using nuclear extracts from TCR/CD3^+ ^uninfected WE17/10 and CD3γ^-^δ^- ^HTLV-I infected WE17/10 cells. *B*, ChIP assay using anti-Sp1, anti-Sp2, anti-Sp3, anti-TFIID, to study the *in vivo *binding to the sequence surrounding the Spγ_**1**_/CD3γ_**Inr **_motif in TCR/CD3^+^uninfected and in CD3γ^-^δ^- ^HTLV-I infected WE17/10 cells.

The h*CD3*γ promoter is lymphoid specific, initiates transcription from multiple start sites, and contains two core promoters capable of recruiting the general transcription machinery through specificity protein (Sp)-binding motifs, with an Initiator (Inr) element present in the primary core promoter[23]. EMSA experiments showed that the complex binding to the Spγ_**1**_/CD3γ_**Inr**_[23] wild-type probe was the same in the nuclear extracts from CD3^+ ^uninfected WE17/10 or from CD3^- ^HTLV-I infected WE17/10 cells (Figure 4A). After HTLV-I infection the *in vitro *binding of transcription factor was apparently not affected in the CD3^- ^HTLV-I infected WE17/10 cells. We analyzed by CHIP the accessibility of the chromatin in the *CD3*γ putative promoter area to the transcriptional machinery after HTLV-I infection. An obvious reduction in accessibility for Sp1, Sp3 and TFIID was observed in CD3^- ^HTLV-I infected WE17/10 cells in comparison with CD3^+ ^uninfected (Figure 4B).

### Treatment with TSA/AZA rescued CD3 mRNA in CD3^- ^HTLV-I infected WE17/10 cells

Treatment of HTLV-I-infected WE17/10 with the histone deacetylase inhibitor (HDACi) trichostatin A in association with the DNA-methylation inhibitor 5' deoxy-azacytidine rescued *CD3*γ and *CD3*δ transcription as assessed by RT-PCR.

**Figure 5:**
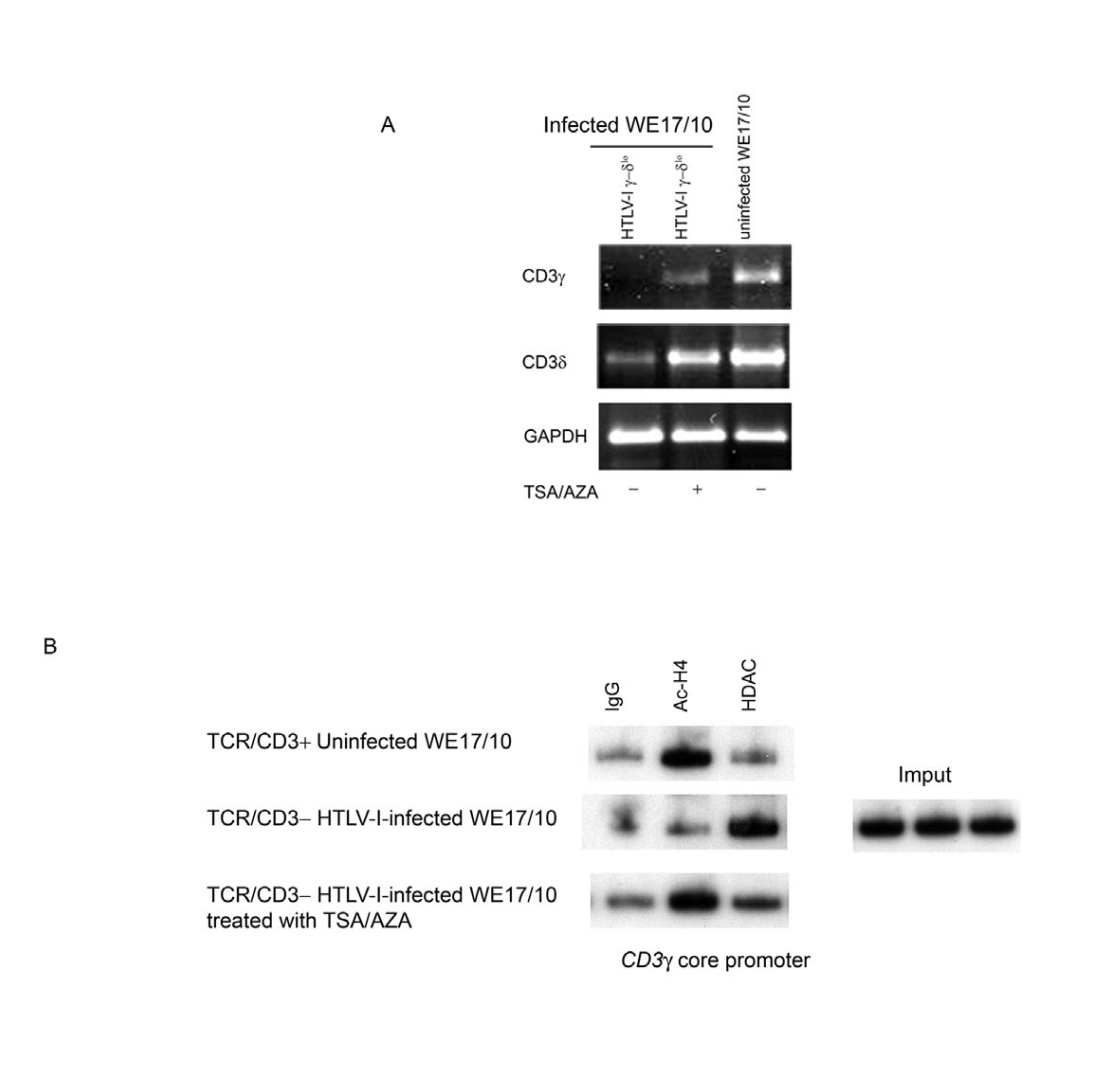
**TSA/AZA treatment of HTLV-I infected WE17/10 cells**. *A*, Representative ethidium bromide-stained gels of *CD3*γ, *CD3*δ and GAPDH (endogenous control) RT-PCR products from untreated HTLV-I infected CD3γ^-^δ^lo^, TSA/AZA HTLV-I infected CD3γ^-^δ^lo ^(treated for 72 hours with 4 μM of 5'AZA and for 18 hours with 500 nM of TSA) and uninfected untreated WE17/10 cells. *B*, ChIP assay using anti-Ac-H4 and anti-HDAC to study the *in vivo *binding to the sequence surrounding the Spγ_**1**_/CD3γ_**Inr **_motif in ^TXP/XΔ3+ ^uninfected and in untreated and TSA/AZA treated CD3γ^-^δ^lo ^HTLV-I infected WE17/10 cells.

Histone H4 hyperacetylation is a typical feature of active transcription; we therefore analyzed chromatin hyperacetylation as well as the binding of HDAC in the *CD3*γ promoter by comparing TCR/CD3^+ ^uninfected, untreated and TSA/AZA treated TCR/CD3^- ^HTLV-I infected WE17/10 cells (Figure 5B). We show that histone hyperacetylation is detectable in CD3^+ ^uninfected WE17/10 cells and TSA/AZA treated CD3^- ^HTLV-I infected WE17/10 cells, but absent in untreated CD3^- ^HTLV-I infected WE17/10 cells. Moreover, *in vivo *binding of HDAC to the *CD3*γ core promoter is more abundant in CD3^- ^HTLV-I infected compared to CD3^+ ^uninfected WE17/10 cells and TSA treated CD3^- ^HTLV-I infected WE17/10 cells.

## Discussion

The T-cell receptor (TCR)/CD3 complex is the keystone of the antigen-specific immune response. Infection by HTLV-I has been shown to ultimately downregulate *CD3*γ, *CD3*δ, *CD3*ε, and *CD3*ζ gene transcripts leading to a CD3^- ^surface phenotype after 200 days of *in vitro *infection[12,13]; however, the sequence of gene loss has not been investigated. We have shown previously that HIV-1 [14-17] and HIV-2[18] associated loss of CD3 expression was characterized by an initial reduction in *CD3*γ gene transcripts. Moreover, infected CD4^+ ^T-cells from patients with ATLL are routinely characterized as having a CD3^- ^or CD3^low ^phenotype [7-9]. The viral load and the natural history of HTLV-I has been studied over 10 years[30] in infected individuals. Interestingly, their figures indicate that HTLV-I+ cells have a very weak contribution to the total number of CD3^+ ^cells. Therefore, it is not surprising that some groups did not find a decrease when looking at the total population of T-cells in patients post HTLV-I infection.

In this study, we investigated proviral integration, viral gene expression, CD3 surface density, CD3 gene transcription and chromatin structure over a period of time of three years post HTLV-I infection of the WE17/10 cell line. We found that HTLV-I *in vitro *infection leads to progressive downmodulation of TCR/CD3 complexes from the cell surface following a pattern of decreasing surface density reminiscent of that observed for HIV-1[14,15] and HIV-2[18], except for its slower kinetics. There is an altered regulation of gene expression affecting initially and more specifically the *CD3*γ gene. To ensure that this phenomenon was not restricted to our experimental setting and the utilized cell line, we have tested a number of well-established HTLV-I infected CD4+ cell lines and found a general down modulation of TCR/CD3 surface expression in comparison to their uninfected counterpart.

However in contrast to the selective targeting of *CD3*γ by HIV[15,18], HTLV-I infection represses in a sequential manner the expression of all four CD3 genes, a distinction obvious at several stages post-infection. Quantification of CD3 gene transcripts in HTLV-I infected cells expressing ~70% of the normal number of surface TCR/CD3 complexes contain only 20% *CD3*γ, 48% *CD3*δ, 62% *CD3*ε and 75% *CD3*ζ gene transcripts. This extensive loss of *CD3*γ transcripts prior to significant TCR/CD3 down-modulation was similar to what we have observed previously for TCR/CD3 loss after HIV-I infection[17]. These data explain why the progression, viewed from the cell surface, appears to be very slow by showing that transcriptional downmodulation is actually initiated early after infection with a considerable and rapid erosion of transcripts until a threshold is reached where the normal number of complete TCR/CD3 complexes can no longer be assembled and exported to the cell surface [31]. Although the complete loss of *CD3*γ parallels the receptor negative phenotype in cell lines infected with both viruses, CD3^- ^HTLV-I infected cells continue to progressively loosing expression of the remaining CD3 genes, with *CD3*δ transcripts being absent at 29 months p.i and about ~25% *CD3*ε and *CD3*ζ transcripts being still expressed at 3 years p.i. In contrast, HIV-1 infected cells maintain *CD3*δ, *CD3*ε and *CD3*ζ transcripts at >75% of normal levels in the presence of steadily decreasing *CD3*γ transcripts. Our data thus reveal that while both HIV-1 and HTLV-I target the expression of the CD3 genes, remarkably they appear to accomplish this task with distinct kinetics.

Importantly, we also observed that, in contrast with HIV infected cells, an in vitro selection of certain clones occurs, as demonstrated in Fig 1, the cells with the lowest CD3 expression growing more rapidly, as we have observed it by comparing the growth speed of cell frozen at different stage of CD3 expression, then put back in culture (data not shown).

The human *CD3*γ, *CD3*δ and *CD3*ε genes, located together on chromosome 11q23, are highly homologous due to their common ancestry[32], while the human *CD3*ζ gene is located on chromosome 1 and has no apparent sequence homology with the other CD3 genes. It is therefore remarkable that all four genes are sequentially targeted in HTLV-I infected cells. Previous studies investigating the role of individual CD3 chains in thymopoiesis suggest that a mechanism exists for controlling access to the *CD3*γ, *CD3*δ and *CD3*ε gene cluster. Disruption of the *CD3*ε gene by insertion of a neomycin cassette in place of either exon 5[30], exons 5 and 6[33] or the promoter plus exons 1 and 2[34] left CD3ε^-/- ^mice who did not only show a *CD3*ε deficiency, but also underwent a significant inhibition of *CD3*γ and *CD3*δ genes transcription. Expression of *CD3*γ and *CD3*δ could be restored in CD3ε^-/- ^mice by deletion of the neomycin cassette using *in vivo *recombination but not by transgenic reconstitution of CD3ε protein expression[35]. Furthermore, insertion of the same neomycin cassette in the contiguous *CD3*γ [36] or *CD3*δ [37] genes had no effect on transcription of their other two neighboring *CD3 *genes. It has been reported that the coding sequence of *neo *gene can act as a transcriptional silencer[38], which suggests that *neo *insertion in *CD3*ε potentially functions as an insulator by separating *CD3*γ and *CD3*δ genes from a putative locus control region. Taken altogether, these data indicate the existence of a mechanism for the global control of the 11q23 CD3 genes cluster that is likely to be critical in modulating the expression of these genes during the early stages of T-cell commitment. Similar cellular factors may also be involved in controlling the *CD3*ζ gene to ensure its coordinate expression with the other CD3 genes during T-cell differentiation and development.

However, by transient transfection we observed that *CD3*γ expression could be restored in HTLV-I infected cells lacking endogenous *CD3*γ expression. This demonstrates that the loss of CD3γ is not due to a defect in factors binding to the CD3γ promoter region and rather suggests a lack of accessibility of these factors to the promoter regions in HTLVI infected cells. We further demonstrated that the loss of *CD3*γ and *CD3*δ transcripts is associated with progressive closure of the *CD3*γ promoter DHS followed by the *CD3*δ promoter and enhancer DHS. Modification in the corresponding DHS occurred in tandem with the reduction and loss of *CD3*γ and *CD3*δ gene expression p.i.

In addition, we showed a reduction *in vivo *binding of Sp1, Sp3 and TFIID to the *CD3*γ core promoter region in CD3^- ^HTLV-I infected WE17/10 cells in comparison with TCR/CD3^+ ^uninfected cells, while the *in vitro *binding was not affected. It has been shown that Sp1 and Sp3 transcription factor binding to TRE-I repeat III participates in the regulation of HTLV-I viral gene expression[39]. On the other hand, epigenetic mechanisms are responsible of HTLV-I-genes transcriptional silencing[40].

Histone H4 hyper-acetylation is a typical feature of active transcription. Histone H4 hyperacetylation was reduced and binding of HDAC to the *CD3*γ core promoter was more abundant in CD3^- ^HTLV-I infected compared to CD3^+ ^uninfected WE17/10 cells. As expected, treatment with the histone deacetylase inhibitor (HDAC) trichostatin A in association with the DNA-methylation inhibitor 5' deoxy-azacytidine reestablished the H4 hyperacetylation status and reduced the HDAC binding to the *CD3*γ core promoter and rescued the transcription of *CD3*γ and *CD3*δ in the CD3^- ^HTLV-I infected. This result reemphasizes that an epigenetic mechanism is at work to downmodulate the four *CD3 *genes after HTLV-I infection. We recently started a study aiming at unraveling the molecular determinants that coordinate the successive downregulation of the four *CD3 *genes.

In a previous work we have shown that HTLV-I infection of WE17/10 CD4^+^cell line leads to progressive alteration of Ca^++ ^influx that eventually results in loss of CD7 expression and activation of an antiapoptotic pathway involving AKT and BAD which paves the way for malignant transformation[10]. Since dysregulation of calcium flux after T-cell activation can be one of the consequences of the lack of TCR/CD3 expression[11] the loss of TCR/CD3 expression could be of significance in the progression of HTLV-1 mediated malignant disease.

## Conclusion

We conclude that HTLV-I expression initiates a process leading to several phenomena, among which a progressive loss of TCR/CD3 by epigenetic mechanisms. These modifications persist after HTLV-I genes are silenced through a mechanism that we have started to investigate. This eventually leads to a CD3^-^, CD7^- ^phenotype associated with perturbation of calcium fluxes and constitutive activation of PI3 kinase, which prevents apoptosis and augments growth of the infected cells. The mechanism by which these phenomena continue after the loss of viral gene expression will be the subject of further studies, as well as determining whether CD3 progressive loss is an epiphenomenon or a causal event in the process of eventual malignant transformation.

## Abbreviations

HTLV-I, human T-cell leukemia virus type I; ATL, adult T cell leukemia/lymphoma; NF-κB, nuclear factor kappa-B; NFAT, nuclear factor of activated T cell; HBZ, HTLV-I bZIP factor; TCR, T cell receptor; HIV, human immunodeficiency virus; DHS, DNase I hypersensitive site; EF-1-α, eukaryotic translation elongation factor1 α; MLN51, cancer susceptibility candidate 3.

## Competing interests

The author(s) declare that they have no competing interests.

## Authors' contributions

HA conceived this project and carried out most of experiments in Figs. 1, 2, 3, 4. BB participated in the design of the study and performed the CHIP experiments. GD carried out the DNase hypersensitivity assays in fig 5. GM participated to the Real time RT-PCR experiments. MMoschitta participated in the constructs of the plasmid used in the transfection assay. MMerimi contributed to the TSA/AZA treatment assay. AB, PM and KW participated in the study design and coordination and helped to draft the manuscript. All authors read and approved the final manuscript.

## Supplementary Material

Additional file 1 CD3 expression on the surface of HTLV-I-infected cells. We have tested the HTLV-I infected cell lines MT-2, C91, WE/HTLV and an ATLL derived cell line PaBe for their TCR/CD3 surface expression. All the cells had a CD3^- ^or CD3^low ^phenotype. Click here for file
