# Supplementary figures and images for: Progressive loss of CD3 expression after HTLV-I infection results from chromatin remodeling affecting all the CD3 genes and persists despite early viral genes silencing
Source: Virol J. 2007 Sep 6;4:85. doi: 10.1186/1743-422X-4-85 (PMC2042505; doi:10.1186/1743-422X-4-85)

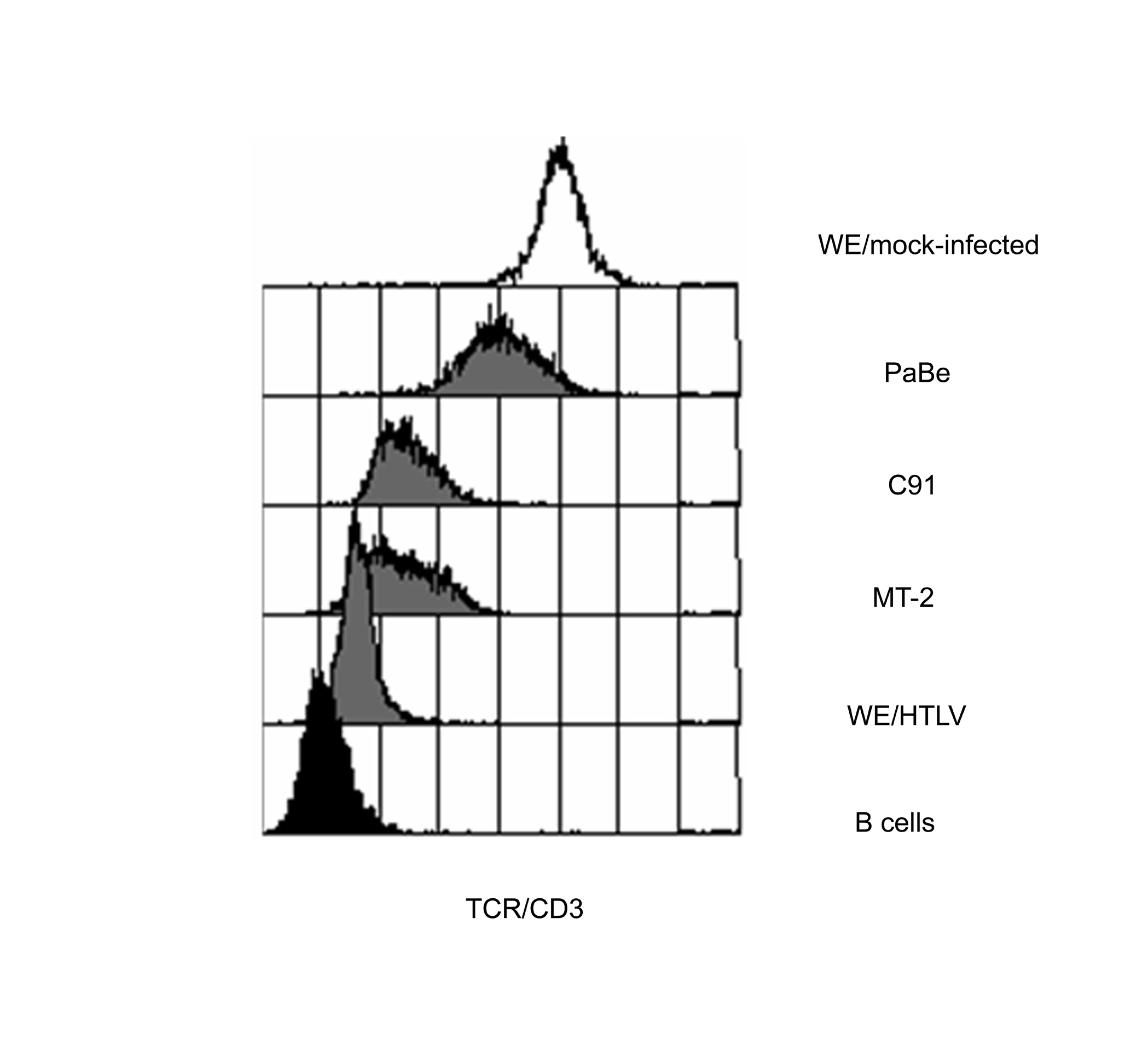

Supplement: Additional file 1 — CD3 expression on the surface of HTLV-I-infected cells. We have tested the HTLV-I infected cell lines MT-2, C91, WE/HTLV and an ATLL derived cell line PaBe for their TCR/CD3 surface expression. All the cells had a CD3- or CD3low phenotype. [file 1743-422X-4-85-S1.png]
